# Pathways Regulating Spheroid Formation of Human Follicular Thyroid Cancer Cells under Simulated Microgravity Conditions: A Genetic Approach

**DOI:** 10.3390/ijms17040528

**Published:** 2016-04-08

**Authors:** Stefan Riwaldt, Johann Bauer, Markus Wehland, Lasse Slumstrup, Sascha Kopp, Elisabeth Warnke, Anita Dittrich, Nils E. Magnusson, Jessica Pietsch, Thomas J. Corydon, Manfred Infanger, Daniela Grimm

**Affiliations:** 1 Plastic, Aesthetic and Hand Surgery, Otto-von-Guericke University Clinic, Leipziger Str. 44, 39120 Magdeburg, Germany; stefan.riwaldt@med.ovgu.de (S.R.); markus.wehland@med.ovgu.de (M.W.); sascha.kopp@med.ovgu.de (S.K.); elisabeth.warnke@med.ovgu.de (E.W.); jessica.pietsch@med.ovgu.de (J.P.); manfred.infanger@med.ovgu.de (M.I.); dgg@biomed.au.dk (D.G.); 2 Max Planck Institute for Biochemistry, Am Klopferspitz 18, 82152 Martinsried, Germany; 3 Institute of Biomedicine, Aarhus University, Wilhelm Meyers Allé 4, 8000 Aarhus C, Denmark; slumstrup@biomed.au.dk (L.S.); adit88@gmail.com (A.D.); corydon@biomed.au.dk (T.J.C.); 4 Medical Research Laboratory, Department of Clinical Medicine, Faculty of Health, Aarhus University, 8000 Aarhus C, Denmark; nm@clin.au.dk

**Keywords:** thyroid cancer, simulated microgravity, random positioning machine, pathway studio, caveolin-1, vascular endothelial growth factor, matrix metalloproteinases, growth

## Abstract

Microgravity induces three-dimensional (3D) growth in numerous cell types. Despite substantial efforts to clarify the underlying mechanisms for spheroid formation, the precise molecular pathways are still not known. The principal aim of this paper is to compare static 1*g*-control cells with spheroid forming (MCS) and spheroid non-forming (AD) thyroid cancer cells cultured in the same flask under simulated microgravity conditions. We investigated the morphology and gene expression patterns in human follicular thyroid cancer cells (UCLA RO82-W-1 cell line) after a 24 h-exposure on the Random Positioning Machine (RPM) and focused on 3D growth signaling processes. After 24 h, spheroid formation was observed in RPM-cultures together with alterations in the F-actin cytoskeleton. qPCR indicated more changes in gene expression in MCS than in AD cells. Of the 24 genes analyzed *VEGFA*, *VEGFD*, *MSN*, and *MMP3* were upregulated in MCS compared to 1*g*-controls, whereas *ACTB*, *ACTA2*, *KRT8*, *TUBB*, *EZR*, *RDX*, *PRKCA*, *CAV1*, *MMP9*, *PAI1*, *CTGF*, *MCP1* were downregulated. A pathway analysis revealed that the upregulated genes code for proteins, which promote 3D growth (angiogenesis) and prevent excessive accumulation of extracellular proteins, while genes coding for structural proteins are downregulated. Pathways regulating the strength/rigidity of cytoskeletal proteins, the amount of extracellular proteins, and 3D growth may be involved in MCS formation.

## 1. Introduction

Altered gravity conditions, such as real or simulated microgravity (µ*g*), offer new and unique approaches to cell biology in general and tissue engineering in particular. Research in Space and experiments using ground-based facilities, such as the fast rotating clinostat (FRC), the random positioning machine (RPM) or rotating wall vessel (RWV) have revealed microgravity-dependent alterations of extracellular matrix proteins and the cytoskeleton, changes in apoptosis, proliferation, and differentiation as well as differences in the growth behavior of human cells [1,2,3,4]. Moreover, microgravity has been shown to promote the scaffold-free growth of 3D multicellular constructs from a monolayer culture of a variety of different cell types, with striking resemblance to actual *in vivo* tissue in several cases [5,6,7].

During the last few years, we have focused on the behavior of malignant thyroid cells [8] under conditions of altered gravity and vibration [9]. We have exposed different healthy and malignant human thyroid cell lines to both real (on parabolic flight campaigns and on the SimBox/Shenzhou-8 Space Mission) and simulated (RPM) µ*g* for different time periods [2,3,4,8,10,11,12,13,14]. These studies showed that healthy and malignant human thyroid cell lines share the capability to form 3D multicellular spheroids (MCS) after exposure to annulled gravity conditions for various time periods [10,11,12]. In general, the cell lines exhibited a similar behavior overall with some differences in detail concerning gene and protein expression as well as the speed of aggregation [2]. The MCS are of great interest for cancer research, because they provide the possibility to study cancer cells *in vitro*. They provide a 3D structure with a known cellular composition. In this state, the cells interact with each other in a 3D way like they do *in vivo*, but differently to being grown within monolayers. In addition, the spheroids′ cells show a much higher invasive potential than monolayer cells [15]. Therefore, by investigating formation and growth of MCS, it might be possible to learn about the formation of metastases and thus to find new, so far neglected targets for cancer therapy/suppression.

Based on the findings of our previous studies, the aim of this study was to further analyze candidate genes and proteins in MCS of human UCLA RO82-W-1 grown on a RPM after 24 h and identify pathways involved in MCS formation by employing both molecular biological (quantitative real-time PCR) as well as *in silico* (Partek analyses) methods.

## 2. Results and Discussion

### 2.1. Spheroid Formation

**Figure 1:**
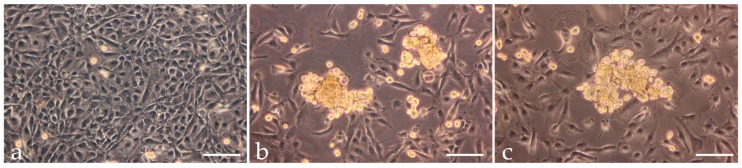
Phase contrast microscopic pictures of UCLA RO82-W-1 1*g*-control cells (**a**), and RO82-W-1 cells cultivated on the Random Positioning Machine (RPM) in slides flasks; (**b**) and T25 cell culture flasks; (**c**) for 24 h. Control cells remained adherent and formed a (sub)confluent monolayer (**a**). In both RPM-samples, some cells remained adherent, while others detached forming 3D spheroids of similar size, thus constituting two distinct cell populations (**b**,**c**). Scale bars = 100 µm.

Phase contrast microscopy revealed that the static control UCLA RO82-W-1 cells grew as a normal monolayer, when the cell culture flasks were placed next to the RPM in the incubator (Figure 1a). However, after a 24 h-exposure of UCLA RO82-W-1 follicular thyroid cancer cells to the RPM, two types of growth were observed: irrespectively of the culture dish used, one part of the cells grew as 3D aggregates, the other part as a two-dimensional monolayer (Figure 1b,c).

This process is well known and has been detected in populations of various types of cells including endothelial cells, chondrocytes, and thyrocytes [6]. It occurs by culturing normal and malignant cells and seems to be due to the effects of microgravity but not to healthy or malignant states of the cells [6,8,14]. Follicle formation can also be induced when the cells are cultured on agarose-coated dishes with a special follicle induction medium [16] or by using the liquid-overlay technique [12]. From these studies, we concluded that a set of proteins interacting properly with each other, drive a cell into a 3D kind of growth. This set is not specific for thyroid cells and does not comprise all proteins changeable under microgravity. For example, thyroid hormones and thyroglobulin are reduced as well as TSH receptors are changed when thyroid cells are cultured under microgravity conditions, while spheroid formation occurs even in their absence [3,17].

**Figure 2:**
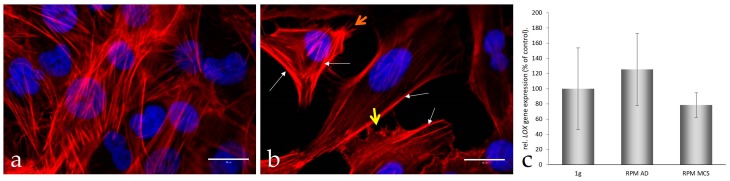
Actin cytoskeleton network visualization and *LOX* gene expression analysis. Rhodamine-phalloidin staining of UCLA RO82-W-1 cells exposed to 1*g* (**a**) and altered gravity on the RPM; (**b**) for 24 h. White arrows designate stress fibers accumulating at the cell borders, the yellow arrow shows lamellipodia and filopodia, the orange one microvilli. Quantitative rtPCR analyses of the *LOX* gene expression in 1*g-*control cells, RPM-adherent cells (AD) and RPM-multicellular spheroids (MCS) cells after a 24 h-culture (**c**). Scale bars = 20 µm (**a** and **b** in bottom right corner).

Moreover, in the adherent part of the RPM-exposed UCLA RO82-W-1 cell stress fibers are visible as well as lamellipodia, filopodia, and microvilli (Figure 2b), which cannot be detected in the corresponding 1*g*-control cells (Figure 2a). This is also in accordance with earlier studies performed on human cells exposed to microgravity, which demonstrated that the cytoskeletal proteins are a preferred target affected by conditions of real and simulated microgravity, irrespectively of whether a cell remains adherent or forms MCS [12,13,18,19].

### 2.2. Impact of Simulated Microgravity on the Activation of Genes Coding for Selected Proteins

**Figure 3:**
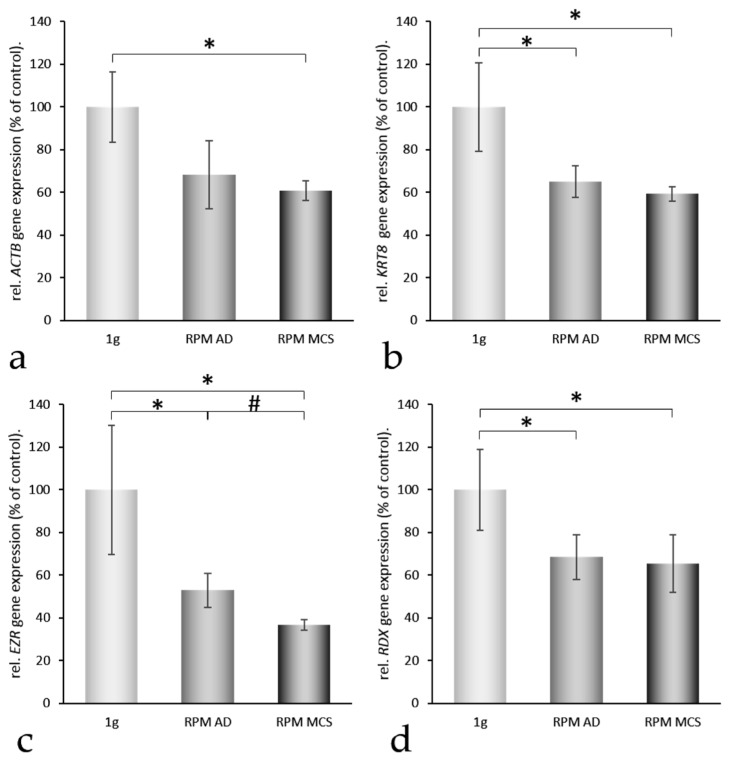

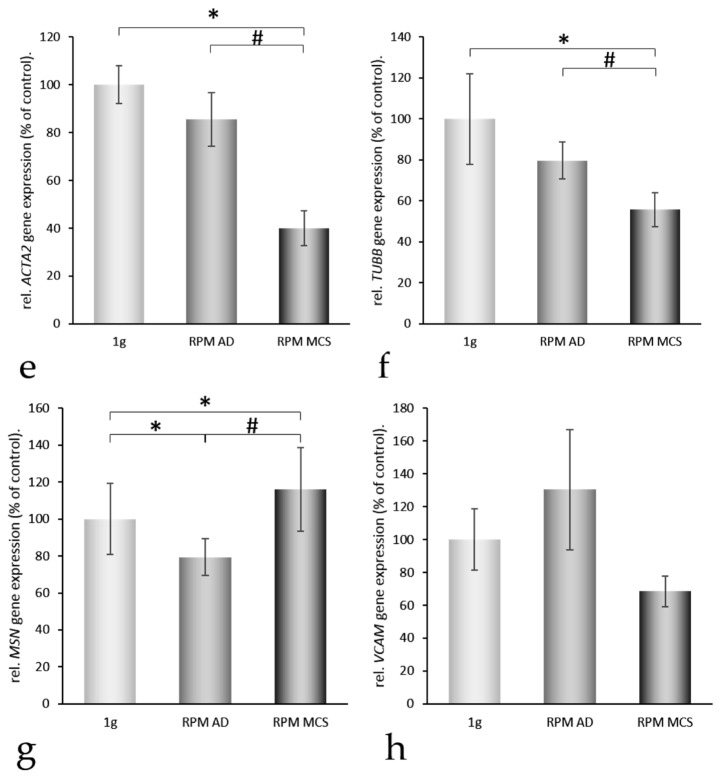
Quantitative real-time PCR of genes of cytoskeletal proteins (Genes of interest I): After 24 h, 1*g*-control, RPM-adherent (AD) and RPM-multicellular spheroids (MCS) were analyzed for their *ACTB* (**a**); *KRT8* (**b**); *EZR* (**c**); *RDX* (**d**); *ACTA2* (**e**); *TUBB* (**f**); *MSN* (**g**); and *VCAM* (**h**) gene expression levels. * = *p* < 0.05 *vs.* 1*g*; # = *p* < 0.05 *vs.* RPM AD.

**Figure 4:**
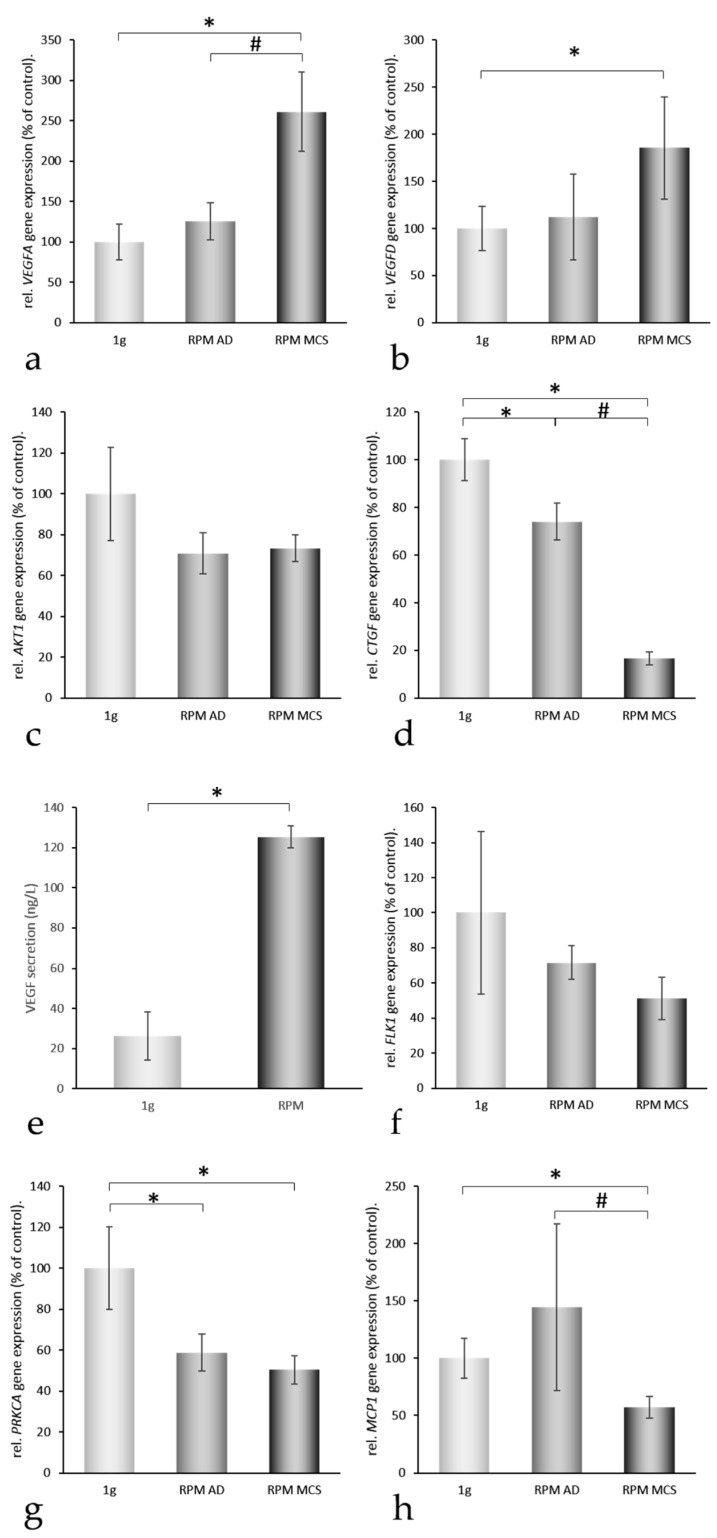
Quantitative real-time PCR of genes of interest II and *VEGF* secretion. After 24 h, 1*g*-control, RPM-adherent (AD) and RPM-multicellular spheroids (MCS) were analyzed for their *VEGFA* (**a**); *VEGFD* (**b**); *AKT1* (**c**); *CTGF* (**d**); *FLK1* (**f**); *PRKCA* (**g**); and *MCP1* (**h**) gene expression levels. In addition, *VEGF* secretion into the culture medium was determined (**e**) * = *p* < 0.05 *vs.* 1*g*; # = *p* < 0.05 *vs.* RPM AD.

**Figure 5:**
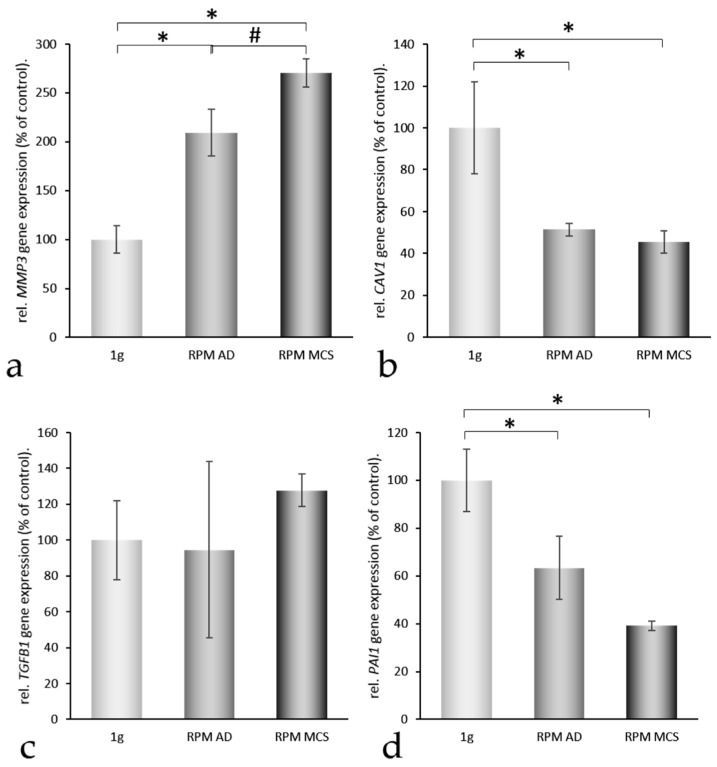

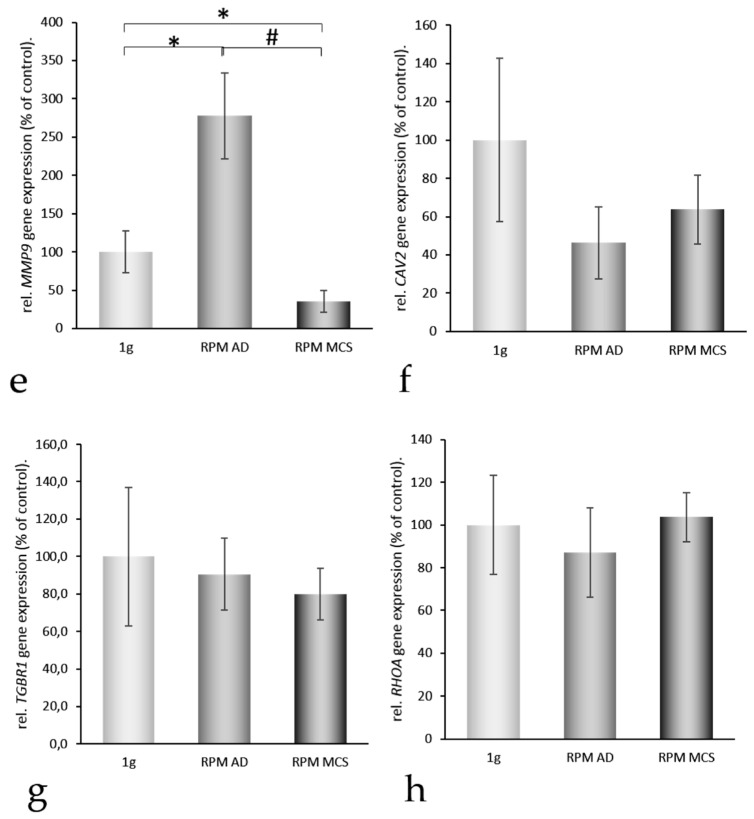
Quantitative real-time PCR of genes of interest III. After 24 h, 1g-control, RPM-adherent (AD) and RPM-multicellular spheroids (MCS) were analyzed for their MMP3 (**a**); CAV1 (**b**); TBFB1 (**c**); PAI1 (**d**); MMP9 (**e**); CAV2 (**f**); TGFBR1 (**g**) and RHOA (**h**) gene expression levels. * = *p* < 0.05 *vs.* 1*g*; # = *p* < 0.05 *vs.* RPM AD.

**Figure 6:**
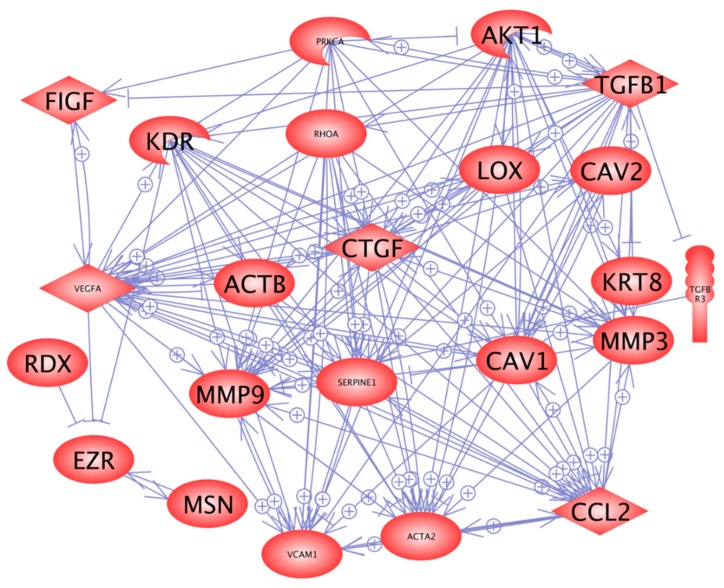
Mutual regulation network at gene expression level. *FIGF*-vascular endothelial growth factor D; *KDR*-kinase insert domain receptor; *SERPINE1*-plasminogen-activator inhibitor-1.

**Figure 7:**
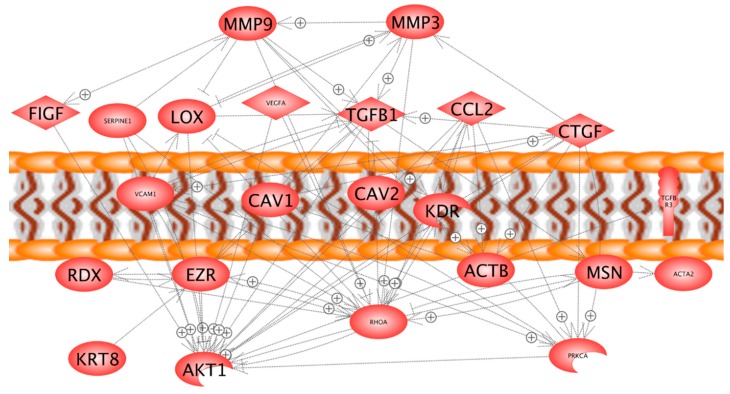
Mutual regulation network at protein level and cellular localization of identified proteins.

In order to find reasons for the transition of the cells from a two- to a three-dimensional kind of growth, we selected 24 genes (Table 1), which attracted attention in our earlier proteomic and genomic studies on spheroid formation of various types of cells [6,18,20]. The genes to be analyzed were selected from a number of preceding publications, where we found that cytoskeletal proteins change, that an overgrowth of extracellular proteins can prevent spheroid formation, that membrane proteins influence this process, and that nuclear proteins are activated [2,4,10,21]. These genes are coding for proteins belonging to three groups: (i) proteins establishing and regulating cell structures (Figure 3a–g); (ii) extracellular proteins regulating the cell behavior (Figure 2c, Figure 4 and Figure 5); and (iii) proteins involved in angiogenesis and signaling processes (Figure 3h, Figure 4 and Figure 5). A Pathway Studio analysis revealed that aside from *TUBB*, the expression of the genes is mutually controlled within the frame of a network (Figure 6). The proteins coded by these genes consisted of five membrane proteins, nine soluble factors, and nine extracellular proteins. They also form a network of regulation, which stretches across the membranes (Figure 7).

In order to examine the impact of an up- or downregulation of a given gene on the other members of the network, we determined the expression of the selected genes in cells exposed to the RPM or cultured under normal 1*g*-conditions. For this purpose, the mRNA expression of the genes was determined by qPCR after they had been cultured on (AD or MCS cells) or next (1*g*-controls) to the RPM for 24 h.

The actin cytoskeleton is involved in adhesion molecule clustering. Therefore, we investigated first the effects exerted by simulated microgravity on the mRNA expression of *VCAM* and cytoskeletal proteins. The gene expression of *ACTB* and *ACTA2* was reduced in MCS and AD cells (Figure 3a,e) compared with 1*g*-controls. In addition, the gene expression of *KRT8* and *TUBB* was downregulated in MCS (Figure 3b,f). Furthermore, the EZR mRNA was clearly decreased in RPM AD samples and further downregulated in MCS compared with 1*g*-samples (Figure 3c). RPM-exposure reduced the *RDX* gene expression significantly in both groups—AD and MCS (Figure 3d), whereas *MSN* decreased in AD, but was elevated in MCS (Figure 3g). There was a tendency of *VCAM* upregulation in AD cells and of a downregulation in MCS (Figure 3h). Taking together the results shown in Figure 3 clearly shows that the expression of the examined genes related to the cytoskeleton was downregulated during spheroid formation early within 24 h

### 2.3. Three-Dimensional Growth Signaling Pathways

Nothing is known about the role of typical angiogenic factors in the early phases of MCS formation. Here we detected a 2.5-fold increase in the *VEGFA* gene expression in MCS samples (Figure 4a) and a 5-fold increase in VEGF protein released in the supernatant (Figure 4e). We could also find an upregulated gene expression of *VEGFD* in MCS samples of UCLA RO82-W-1 cells (Figure 4b). The *VEGFR2* (*FLK1*) mRNA appeared to be slightly although not significantly downregulated in RPM-samples after 24 h (Figure 4f). The *AKT1* gene expression remained unchanged (Figure 4c). Focusing on the protein kinase C (PKC) pathway, which is involved in cell proliferation and is affected during spheroid formation [10], we could measure a clear reduction of *PRKCA* mRNA after RPM-exposure in AD and MCS samples compared with corresponding static 1*g*-controls (Figure 4g). As a representative member of growth factors influencing cell proliferation we investigated possible changes in the connective tissue growth factor (*CTGF*) gene expression. After a 24 h-RPM-exposure, *CTGF* mRNA was downregulated (Figure 4d). Monocyte Chemoattractant Protein-1 (MCP-1) had been identified to play an important role in 3D formation under conditions of weightlessness [21]. The *MCP1* gene expression was downregulated in MCS (Figure 4h), but remained unchanged in AD cells. Hence Figure 4 shows that two of the angiogenic-related genes (*VEGFA, VEGFD*) after RPM-exposure of the cells were upregulated.

We previously have shown that genes and proteins involved in the regulation of FTC-133 thyroid cancer cell proliferation and metastasis, such as VEGFA and VEGFD were similarly regulated under long-term RPM and spaceflight conditions (10 days microgravity) [4]. After 10 days in Space, we detected a reduced *VEGFA* mRNA in RPM AD cells and MCS cultures, as well as spaceflight AD cells and MCS cultures [4]. Interestingly, the *VEGFD* mRNA was increased in the RPM and in Space cultures of the Shenzhou-8 Space Mission [4], which is comparable to the 24 h-result in UCLA RO82-W-1 RPM samples.

### 2.4. Factors Regulating the Amount of Extracellular Proteins

We described recently that the quantities of extracellular matrix (ECM) proteins play a decisive role in spheroid formation [22]. An increased ECM together with enhanced amounts of Caveolin-1, which scaffolds several proteins like PKC or KDR within the membrane [21,22] can cause a firm anchoring of the cells within the ECM and inhibits spheroid formation [22].

Therefore, we also studied gene alterations of the matrix metalloproteinases MMP3 and MMP9. They are known to play a role in regulating the amount of extracellular matrix proteins [23,24]. Both, *MMP3* and *MMP9* mRNAs were significantly elevated in AD cells (Figure 5a,e). *MMP3* was further enhanced in MCS compared with 1*g*-controls, whereas *MMP9* was blunted in MCS samples (Figure 5a,e). Caveolin-1 and -2 were both expressed by UCLA RO82-W-1 cells. The *CAV1* mRNA was downregulated in AD and MCS (Figure 5b). A similar result was obtained for *CAV2* (Figure 5f). Both *TGFB1* and *TGFBR1* gene expression remained unaltered (Figure 5c,g).

Recent pathway analyses demonstrated that enhanced concentrations of plasminogen might inhibit spheroid formation [21,22]. We measured the plasminogen activator inhibitor-1 (*PAI1*) gene expression. *PAI1* was significantly downregulated in AD and MCS samples (Figure 5d), which may favor the degradation of extracellular plasminogen [25]. In addition, the *RHOA* mRNA was not altered after a 24 h-RPM-exposure (Figure 5h).

### 2.5. In Silico Search for Mutual Gene Regulation

Figure 3, Figure 4 and Figure 5 indicated that cells remaining adherent during the early 24 h of incubation kept 16 of the 24 genes investigated within the frame of not-significant variation, downregulated seven, and upregulated one. In contrast, cells growing three-dimensionally exerted three upregulated genes, downregulated 11, and kept only 10 within the frame of not-significant variation. In order to find reasons how the changes of the genes mentioned above may contribute to a transition from a two- to three-dimensional growth behavior of UCLA RO82-W-1 follicular thyroid cancer cells, we wanted to find relationships between the networks shown in Figure 6 and Figure 7 and the gene expression changes indicated in Figure 3, Figure 4 and Figure 5. The most significant upregulation was indicated for the *VEGFA* and *VEGFD* genes only in MCS cells (Figure 4a,b). A simultaneous upregulation of both genes had already been observed in de-differentiated human ovarian carcinomas [26]. Regarding *VEGFA*, not only the gene was upregulated, but also the quantities of VEGFA proteins released in the supernatant were enhanced (Figure 4e). This indicates that both AD cells and MCS cells grew on the RPM under enhanced concentrations of external VEGFA. However, only those cells, showing an upregulated *VEGFA* mRNA in combination with an elevated *VEGFD* mRNA after the first 24 h of RPM-exposure, formed spheroids (Figure 1).

In our system, *i.e.*, in the presence of enhanced external VEGFA protein, the MCS-cells with enhanced simultaneous *VEGFA* and *VEGFD* mRNA expression showed a significant downregulation of the α/β-actin expression, while the AD cells kept their actin expression rates similar to the 1*g*-control cells. It has already been described that VEGF downregulates α/β-actin expression in retinal endothelial cells or in arterial smooth muscle cells after addition of external VEGFA [27,28]. The mechanism is unknown, but could include miR-205 and *EZR* mRNA production, as described by Li *et al.* [29].

The *MMP3* expression is upregulated in AD cells and even more in MCS cells (Figure 5a). This corresponds to the observation of Saleh *et al.* who showed that the *MMP3* gene expression in peripheral blood mononuclear cells is depending on their own expression of the *VEGF* gene as well as on plasma VEGF concentration [30]. In addition, *MMP9* is upregulated (Figure 5e). *VEGF* is known to upregulate the expression of MMP9 in lung macrophages as long as a VEGFR-1 receptor is present on the surface [31]. The *MMP9* expression may also be upregulated by MCP1 [32]. Its expression is elevated in AD cells, but lowered in MCS cells (Figure 5e).

Furthermore, the *PAI-1* (Figure 5d) gene expression was downregulated in the presence of the enhanced VEGF, while *TGFB1* (Figure 5c) gene expression remained unchanged. A similar VEGF effect was described for endothelial cells [33]. We recently found that plasminogen accumulation contributes to inhibition of spheroid formation [22]. Hence, an attenuation of the inhibition of plasminogen activators due to downregulation of *PAI-1* could help to form spheroids [34].

Although it has been demonstrated in a number of studies that VEGF upregulates the FLK1 [34], we observed a slight but insignificant downregulation of the mRNA of *FLK1* (Figure 4f). This might be explained by a decreased amount of *CAV1*, which scaffolds FLK1 [21,35] or by the presence of various VEGF-A isoforms. For example, the VEGF-A121 was found to have little effect on KDR expression [36]. Similarly, the *VCAM* expression depends on the isoforms of the VEGF surrounding the cells [37].

Furthermore, the *LOX* (monoamine oxidase lysyl oxidase) expression was not significantly changed under the conditions of elevated *VEGF* in microgravity (Figure 2c), although in ARPE-19 cells a VEGF-dependent increase of enzyme activity as well as the mRNA expression of *LOX* was found [38]. Interestingly, a slight but insignificant increase of *LOX* mRNA was found in AD cells, while *LOX* was simultaneously decreased in MCS cells (Figure 2c). This is in accordance to our earlier hypothesis that a removal of LOX favors spheroid formation [39]. It is known that LOX is involved in several steps of metastasis and it might be an interesting protein to be investigated in co-culture experiments with endothelial cells in the future.

It is described in the literature that VEGF upregulates *CCL-2* as well as the *CTGF* gene expression [40,41]. In AD cells we found that *CTGF* was slightly downregulated, but *MCP1* is upregulated insignificantly though. In MCS cells both types of genes were further downregulated. This result suggests that AD and MCS respond differently to VEGF.

This study was designed to investigate the underlying mechanisms for 3D growth on the RPM. The study revealed that during exposure to microgravity the UCLA RO82-W-1 cell population splits in adherent and three-dimensionally growing cells. This observation fits to results obtained by others as well as by our group. For example, when cultured on the RPM murine osteoblasts and human breast cancer cells split into two populations with different phenotypes, respectively [42,43]. We previously described malignant FTC-133 thyroid cancer cells, healthy chondrocytes, or endothelial cells growing in one culture flask simultaneously in a two- or a three-dimensional manner [2,3,6,8,44].

Interestingly, only seven of the 24 genes investigated showed significantly different gene expression levels in AD and MCS cells. This finding suggests that neighboring cells of one culture change distinct genes in different ways. Attempts were made to explain the split of cell population into different sub-populations, stressing the phenomenon of bifurcation [43,45,46,47]. The authors of this hypothesis suggest that the cells, when exposed to microgravity, come into a state in which very small secondary effects, such as gene variations observed in single cell transcriptomes of a seemingly homogenous population, may trigger the development of single cells into different directions [48]. In our earlier experiments, flow cytometry measurements repeatedly indicated considerable variations of the number of distinct antigens at the cell surfaces. Cell electrophoretic studies showed that the overall surface charge density varies from cell to cell in a way that two distinct cell populations emerge [49,50]. In addition, we found that the glycolytic enzyme alpha-enolase was differently expressed in different cells of a population [51]. Therefore, the task remains to examine the accumulation of extracellular proteins, whose overexpression prevent spheroid formation in a whole culture, in regard to individual cells and to study whether different accumulation of the proteins described recently could prevent spheroid formation on an individual cell basis [22]. This way, we will learn whether MCS cells stem from those cells being driven out from the monolayer by microgravity but not by normal gravity, because of their lower accumulation of extracellular matrix proteins than that of their neighbors.

In accordance with earlier investigations performed on human follicular thyroid cells exposed to microgravity, these experiments also suggest that the cytoskeletal proteins and their organizers ezrin, radixin, and moesin [52] are a preferred target affected by conditions of real and simulated microgravity [13,19]. Together they organize and maintain the cell cortex as well as the interface between the extracellular environment/cell membrane, the cytoskeleton, and cytoplasm. They achieve this by interactions with both membrane and filamentous actin or by linking receptors to further signaling components. Moreover, they are involved in the regulation of several signaling pathways [53]. Besides moesin, the genes of all analyzed cytoskeletal proteins were downregulated. As one can assume that both adherent and MCS cells are exposed to similar mechanical forces, it seems reasonable that mechanical forces are not the only factors, affecting the cytoskeleton. However, mechanical forces could be more effective in changing the cell’s phenotype, if the quantity of actin or ezrin is reduced [54,55,56].

α and β actins as well as ezrin, which are cytoskeletal key factors, might be downregulated by VEGF. The most interesting result was that *VEGFA* and *VEGFD* were significantly upregulated only in MCS cells. We do not know the causes of the VEGF upregulation, as all the factors shown in Figure 6 ((+) sign on arrow) to favor upregulation of *VEGF* remained in RPM-exposed cells either similar (*AKT-1*, *TGFB1*, *RHOA*) or were significantly downregulated (*PAI-1*, *MCP-1*) as compared to the 1*g*-controls. Interestingly, *CTGF* was downregulated in AD cells by 25% and in MCS cells by 85%. A downregulation of *CTGF* may enhance the bioavailability of VEGF within the spheroids, because CTGF binding can neutralize VEGF activity [57]. VEGF regulates vascularization in wound healing by challenging outgrowth of endothelial cells [58], but also has a great influence on cancer development and metastasis [59]. It is known that an increase in *VEGFA* indicates neoangiogenesis, low-differentiation and progression in cancer [59,60]. In addition, VEGF production is modified, when cells grow under microgravity [61]. We had recently demonstrated that microgravity-exposure of FTC-133 thyroid cancer cells for 10 days in Space and on the RPM induced a downregulation of VEGFA, but an upregulation of VEGFD [4]. VEGFA and VEGFD exert differently strong effects on their target cells and by competing for the equal receptor-binding site VEGFD may fine-tune the VEGFA activities [62]. Hence, both VEGFs seem to play different roles in wound healing, tumor progression and microgravity-induced reactions. In 2012 Nersita *et al.* [63] reported a low level of VEGFD in metastatic thyroid carcinomas, which may indicate that VEGFA effects are not regulated anymore [63].

It was demonstrated in human endothelial cells (EA.hy926 cell line) that these cells also produced more than normal amounts of VEGF and FLK-1 by themselves [44]. VEGF was increased after a 4 h-RPM-exposure and was further elevated after 12 h [44]. In addition, an RPM-exposure of the EAhy926 endothelial cells early induced FLK-1 protein (within 4 h). This increase was further elevated after a 12 h-RPM-exposure [44]. Interestingly, the external application of 10ng/mL VEGF to the culture medium reduced the amount of synthesized VEGF protein [44]. Furthermore, EAhy926 cells incubated for seven days either under 1*g*-conditions or under s-μ*g* revealed a clear increase in the VEGF expression of RPM-exposed cells compared with 1*g*-controls [61]. In this long-term experiment, it could be demonstrated that external VEGF (10 ng/mL) did not induce a further elevation of VEGF [61]. Gravitational unloading alone stimulated the endothelial cells to synthesize VEGF early. The EA.hy926 cell line is a human umbilical vein cell line, established by fusing primary human umbilical vein cells with a thioguanine-resistant clone of A549 lung carcinoma cells by exposure to polyethylene glycol [64]. Cancer cells are able to fuse spontaneously with endothelial cells to form hybrid cells, facilitating the cells traversing the endothelial barrier to form metastases [65]. These characteristics will help to increase our knowledge in the processes of angiogenesis or metastasis with the aim of finding future drug targets for cancer therapy. In future studies, it is a matter of interest to investigate the impact of conditioned medium of microgravity-related cancer cells on endothelial cells or by a co-culture assay.

*MMP3* had shown to be upregulated under microgravity. The *MMP3* gene product can have several functions. First, it may be located within the nucleus of cells like chondrocytes and activate transcription enhancers, which may e.g., in chondrocytes activate the transcription of *CTGF* genes [66]. This pathway seems not to be active in our system, because the *CTGF* gene is downregulated at enhanced transcription of *MMP3* (Figure 4 and Figure 5). It is more probable that MMP3 degrades the VEGFR or digests products of the extracellular matrix [67]. In a recent spaceflight experiment, we found that an elevated protein accumulation in the extracellular space could detain 3D formation, while profilin-1 was phosphorylated in Space [22]. Hence, elevated MMP3 may favor the degradation of proteins surrounding the cells and in this way support spheroid formation.

CTGF is involved in the spheroid formation of FTC-133 thyroid cancer cells in Space [11]. The *CTGF* mRNA expression was enhanced by annulling gravity in space-flown samples, suggesting a key role of this growth factor in continuing 3D growth type [11]. A reduced *CTGF* mRNA expression in 3D aggregates compared to adherent cells was observed on the two devices [68]. *In vitro*, CTGF has been shown to stimulate ECM production, chemotaxis, proliferation, and integrin expression and has been implicated in various biological processes, such as cell proliferation, migration, angiogenesis, and tumorigenesis [69]. It has also been shown that *CTGF* expression level was elevated in primary papillary thyroid carcinoma samples and was correlated with clinical features, such as metastasis, tumor size, or the clinical stage [70]. After 24 h on the RPM, *CTGF* was downregulated in UCLA RO82-W-1 cells. Therefore, the importance of *CTGF* in spheroid formation of thyroid cancer cells has to be further investigated in the future.

*In vitro*, the protein kinase C-α (PRKCA) is involved in the control of human medullar thyroid carcinoma cell proliferation and survival by modulating apoptosis [71]. *PRKCA* gene expression was downregulated by vibration in human thyroid cells. In this experiment, it was also downregulated. A factor triggering this downregulation could not be seen in our experiments. However, it is known that PRKCA initiates upregulation of *CAV1* or *CTGF* [72,73]. Therefore, one might conclude that *PRKCA* expressed as found in our experiments may not be capable to enforce upregulation of *CAV1* or *CTGF* genes [21]. It appears worthwhile to study the PRKCA effects on *CAV1* expression because of the capability of CAV1 to inhibit the cellular sheering out of a monolayer in cancer cells [21].

## 3. Experimental Section

### 3.1. Cells

The UCLA RO82-W-1 cell line was used in this study and purchased from Sigma-Aldrich Chemie (Munich, Germany). The cell line was derived from the metastases of a follicular carcinoma in a female patient. The primary tumor released thyroglobulin (>10,000 ng/mL), but the uptake of I^131^ was less than 2%. Immunoperoxidase staining revealed thyroglobulin-positivity within the cells. The cell line was tumorigenous in nude mice [74]. The cells were cultured in RPMI-1640 medium containing 100 μM sodium pyruvate and 2 mM·l-glutamine, supplemented with 10% fetal calf serum (FCS), 100 U/mL penicillin and 100 µg/mL streptomycin (all Invitrogen, Eggenstein, Germany).

### 3.2. Random Positioning Machine

30 T25 culture flasks were seeded with 1 × 10^6^ UCLA RO82-W-1 cells each and incubated over night at 37 °C and 5% CO_2_ in an incubator to ensure attachment of the cells to the bottom of the culture flask. The next day, the flasks were completely filled with medium carefully avoiding air bubbles. 15 T25 were fixed on the RPM, as close as possible to the center of the platform, which was then rotated at a speed of 60 °/s in real random mode. The RPM was positioned in a commercially available incubator set at 37 °C and supplied with 5% CO_2_. 15 T25 culture flasks for 1*g*-ground control cultures grown in parallel in identical equipment were kept statically in the same incubator as the RPM.

### 3.3. F-Actin Staining

UCLA RO82-W-1 cells were seeded into slide flasks (BD, Heidelberg, Germany) and placed in an incubator (37 °C, 5% CO_2_) overnight, until they attached to the slides. The next day, the slide flasks were completely filled with medium avoiding air bubbles, sealed with parafilm, and placed on the RPM for 24 h. F-actin was visualized by means of rhodamine-phalloidin staining (Molecular Probes^®^, Eugene, OR, USA). The method was described earlier in detail [75,76].

### 3.4. RNA Isolation

Directly after the experiments, the cells were fixed with RNA*later* (Thermo Fisher Scientific, Roskilde, Denmark). For harvesting of the cells, RNA*later* was replaced by PBS (Invitrogen, Darmstadt, Germany). The method was published recently in detail [75,77].

### 3.5. Quantitative Real Time PCR

We employed the real-time quantitative RT PCR to quantify expression levels of the genes of interest. Appropriate primers with a Tm of about 60 °C were designed with the Primer Express^®^ (Version 2.0.0, Applied Biosystems, Foster City, CA, USA) software. The primers were synthesized by TIB Molbiol (Berlin, Germany) and listed in Table 1. The method was published recently in detail [75,77].

### 3.6. Pathway Studio Analysis

Pathway Studio v11 was purchased from Elsevier Research Solutions, Amsterdam, The Netherlands. This program was used online [78]. To start an analysis, the SwissProt numbers of the proteins of interest were entered.

### 3.7. Statistics

All statistical analyses were performed using SPSS 21.0 (SPSS, Inc., Chicago, IL, USA, 2012). The data was analyzed with the Mann–Whitney *U* test. To account for multiple comparisons, a Kruskal–Wallis Test was performed beforehand, and Bonferroni corrections were applied. The data was expressed as means ± standard deviation (SD). Differences were considered significant at *p* < 0.05.

## 4. Conclusions

This is the first study, using pathway analyses programs to investigate the molecular mechanisms responsible for 3D growth of follicular thyroid cancer cells grown under conditions of simulated microgravity for 24 h on the RPM. Earlier studies have shown, that human and rat benign thyroid cells as well as cancer cells *in vitro* and *in vivo* respond to microgravity conditions and induce a variety of changes in these cells [3,8,17,79,80,81,82,83,84,85,86]. This response might give important hints for cancer research on Earth [87].

The appearance of the two distinct cell populations, adherent cells and MCS, fits very well to the phenomenon of the bifurcation point. It remains unclear if initially a shift in the phenotype leads to a change in gene expression or if the differential gene expression leads to the shift in the phenotype, but it is very certain that both can influence each other. We still do not know, which one of the differentially expressed genes is the very first step triggered by the removal of gravity. However, we recognized, that in the cells with an upregulated *VEGFA* and *VEGFD* mRNA, spheroid formation could be favored, because upregulated *MMP-3* mRNA and simultaneously downregulated *PAI1* mRNA favor the degradation of the extracellular proteins and reduction of actin and ezrin proteins. These changes may facilitate a transition of the phenotypes, while the *PRKCA* mRNA expression is too low to enforce upregulation of *CAV-1* and *MCP1*.

## Figures and Tables

**Table 1:** Primer used for quantitative real-time PCR.

Gene	F-Primer	Sequence	R-Primer	Sequence
*18S rRNA*	18S-F	GGAGCCTGCGGCTTAATTT	18S-R	CAACTAAGAACGGCCATGCA
*ACTA2*	ACTA2-F	GAGCGTGGCTATTCCTTCGT	ACTA2-R	TTCAAAGTCCAGAGCTACATAACACAGT
*ACTB*	ACTB-F	TGCCGACAGGATGCAGAAG	ACTB-R	GCCGATCCACACGGAGTACT
*AKT1*	AKT1-F	CTTCTATGGCGCTGAGATTGTG	AKT1-R	CAGCATGAGGTTCTCCAGCT
*CAV1*	CAV1-F	CCTCCTCACAGTTTTCATCCA	CAV1-R	TGTAGATGTTGCCCTGTTCC
*CAV2*	CAV2-F	GATCCCCACCGGCTCAAC	CAV2-R	CACCGGCTCTGCGATCA
*CTGF*	CTGF-F	ACAAGGGCCTCTTCTGTGACTT	CTGF-F	GGTACACCGTACCACCGAAGAT
*EZR*	EZR-F	GCAATCCAGCCAAATACAACTG	EZR-R	CCACATAGTGGAGGCCAAAGTAC
*FLK1*	FLK1-F	TCTTCTGGCTACTTCTTGTCATCATC	FLK1-R	GATGGACAAGTAGCCTGTCTTCAGT
*KRT8*	KRT8-F	GATCTCTGAGATGAACCGGAACA	KRT8-R	GCTCGGCATCTGCAATGG
*LOX*	LOX-F	TGGGAATGGCACAGTTGTCA	LOX-R	AGCCACTCTCCTCTGGGTGTT
*MCP1*	MCP1-F	GCTATAGAAGAATCACCAGCAGCAA	MCP1-R	TGGAATCCTGAACCCACTTCTG
*MMP3*	MMP3-F	ACAAAGGATACAACAGGGACCAA	MMP3-R	TAGAGTGGGTACATCAAAGCTTCAGT
*MMP9*	MMP9-F	CCTGGAGACCTGAGAACCAATC	MMP9-R	TTCGACTCTCCACGCATCTCT
*MSN*	MSN-F	GAAATTTGTCATCAAGCCCATTG	MSN-R	CCATGCACAAGGCCAAGAT
*PAI1*	PAI1-F	AGGCTGACTTCACGAGTCTTTCA	PAI1-R	CACTCTCGTTCACCTCGATCTTC
*PRKCA*	PRKCA-F	TGGGTCACTGCTCTATGGACTTATC	PRKCA-R	CGCCCCCTCTTCTCAGTGT
*RDX*	RDX-F	GAAAATGCCGAAACCAATCAA	RDX-R	GTATTGGGCTGAATGGCAAATT
*RHOA*	RHOA-F	CGTTAGTCCACGGTCTGGTC	RHOA-R	GCCATTGCTCAGGCAACGAA
*TGFB1*	TGFB1-F	CACCCGCGTGCTAATGGT	TGFB1-R	AGAGCAACACGGGTTCAGGTA
*TGFBR1*	TGFBR1-F	CGCACTGTCATTCACCATCG	TGFBR1-R	CACGGAACCACGAACGTTC
*TUBB*	TUBB-F	CTGGACCGCATCTCTGTGTACTAC	TUBB-R	GACCTGAGCGAACAGAGTCCAT
*VCAM*	VCAM-F	CATGGAATTCGAACCCAAACA	VCAM-R	GGCTGACCAAGACGGTTGTATC
*VEGFA*	VEGFA-F	GCGCTGATAGACATCCATGAAC	VEGFA-R	CTACCTCCACCATGCCAAGTG
*VEGFD*	VEGFD-F	TGCAGGAGGAAAATCCACTTG	VEGFD-R	CTCGCAACGATCTTCGTCAA

*ACTA2*: α-actin-2; *ACTB*: Actin β; *AKT1*: Nuclear factor NF-κ-B activator 1; *CAV1*: Caveolin 1; *CAV2*: Caveolin 2; *CTGF*: Connective tissue growth factor; *EZR*: Ezrin; *FLK1*: Vascular endothelial growth factor receptor 2; *KRT8*: Cytokeratin-8; *LOX*: Oxidized low-density lipoprotein receptor 1; *MCP1*: Monocyte chemotactic protein 1; *MMP3*: Matrix metalloproteinase-3; *MMP9*: Matrix metalloproteinase-9; *MSN*: Moesin; *PAI1*: Plasminogen activator inhibitor 1; *PRKCA*: Protein kinase C alpha type; *RDX*: Radixin; *RHOA*: Ras homolog gene family, member A; *TGFB1*: Transforming growth factor β-1; *TGFBR1*: TGF-β receptor type-1; *TUBB*: Tubulin β, *VCAM*: Vascular cell adhesion protein 1; *VEGFA*: Vascular endothelial growth factor A; *VEGFD*: Vascular endothelial growth factor D; F: Forward; R: Reverse. All sequences are given in 5′–3′ direction.

## Abbreviation

ACTA2	Actin aortic smooth muscle (P62736)
ACTB	Actin cytoplasmic 1 (P60709)
AD	Adherent
AKT1	RAC-α serine/threonine-protein kinase (P31749)
CAV1	Caveolin-1 (Q03135)
CAV2	Caveolin-2 (P51636)
CCL2	C–C motif chemokine 2 (P13500)
CTGF	Connective tissue growth factor (P29279)
EZR	Ezrin (P15311)
FIGF or VEGFD	Vascular endothelial growth factor D (O43915)
FLK1 or KDR	Vascular endothelial growth factor receptor 2 (P35968)
KRT8	Keratin type II cytoskeletal 8 (P05787)
LOX	Protein-lysine 6-oxidase (P28300)
MCS	Multicellular spheroids
MCTS	Multicellular tumor spheroid
MMP-3	Matrix metalloproteinase-3 or Stromelysin-1 (P08254)
MMP-9	Matrix metalloproteinase-9 (P14780)
MSN	Moesin (P26038)
PRKCA	Protein kinase C alpha type (P17252)
qPCR	Quantitative real-time PCR
RDX	Radixin (P35241)
RHOA	protein: Ras homolog gene family, member A; gene: Transforming protein RhoA (P61586)
RPM	Random Positioning Machine
RWV	Rotating Wall Vessel
SERPINE1	Plasminigen activator inhibitor 1 (P05121)
TGFB1	Transforming growth factor β-1 (P01137)
TGFBR1	Transforming growth factor beta receptor type 1 (P36897)
TGFBR3	Transforming growth factor beta receptor type 3 (Q03167)
TUBB	Tubulin β (P07437)
VCAM1	Vascular cellular adhesion protein 1 (P19320)
VEGFA	Vascular endothelial growth factor A (P15692)
